# Histopathological analysis of a retrieved cerebral thrombus revealing occult lung adenocarcinoma: a case report

**DOI:** 10.1186/s13256-026-06487-w

**Published:** 2026-08-20

**Authors:** Carlota Jauregui-Larrañaga, Inés Albajar, María Aranzazu Juaristi-Abaunz, Juan Marta-Enguita

**Affiliations:** 1 Department of Neurology, Biogipuzkoa Health Research Institute, Donostia University Hospital, Paseo Dr. Begiristain s/n, Begiristain Doktorea Pasealekua, 20014 San Sebastián-Donostia, Guipúzcoa Spain; 2 Department of Neurology, Donostia University Hospital, Paseo Dr. Begiristain s/n, 20014 San Sebastián-Donostia, Guipúzcoa Spain; 3 Department of Anatomical Pathology, Donostia University Hospital, Paseo Dr. Begiristain, 20014 San Sebastián-Donostia, Guipúzcoa Spain; 4 Valdecilla Health Research Institute (IDIVAL), Calle Cardenal Herrera Oria, 39011 Santander, Cantabria Spain; 5 Department of Neurology, Marqués de Valdecilla University Hospital, Avenida de Valdecillas/n, 39008 Santander, Cantabria Spain

**Keywords:** Stroke, Cancer-associated stroke, Tumour embolism, Mechanical thrombectomy, Lung cancer, Adenocarcinoma, Thrombus histopathology

## Abstract

**Background:**

Cancer-associated stroke (CAS) is a heterogeneous entity in which hypercoagulability represents the most common mechanism, whereas direct tumour embolism is rare and likely under-recognised.

**Case presentation:**

A 76-year-old white European man presented with acute left hemiplegia, hemianopsia and dysarthria (NIHSS 18). Non-contrast CT showed early ischaemic changes (ASPECTS 7), CT angiography demonstrated occlusion of the right middle cerebral artery, and CT perfusion revealed a favourable mismatch profile. Mechanical thrombectomy achieved partial recanalisation (TICI 2b). Histopathological examination of the retrieved thrombus revealed malignant epithelial cells. Immunohistochemical analysis was consistent with lung adenocarcinoma with high PD-L1 expression. Continuous cardiac monitoring and 72-h Holter-ECG showed no atrial fibrillation, and transthoracic echocardiography identified no major cardioembolic source. Laboratory testing showed only moderately elevated D-dimer levels. Subsequent chest CT identified a large necrotic mass in the left lung invading the pulmonary vein. Despite initiation of immunotherapy, the patient developed brain metastases and died 51 days after the index stroke.

**Conclusions:**

Tumour embolism is a rare but clinically relevant mechanism of ischaemic stroke. It should be considered in patients with embolic stroke of undetermined source, even in the absence of markedly elevated D-dimer levels. Histopathological analysis of thrombectomy specimens may provide clinically relevant diagnostic information and facilitate early detection of occult malignancy in selected patients.

## Background

Cancer-associated stroke (CAS) is increasingly recognised as a complex clinical entity involving several pathogenic mechanisms, including hypercoagulability, non-bacterial thrombotic endocarditis (NBTE), direct vascular invasion and treatment-related vascular toxicity [1]. Hypercoagulability represents the predominant mechanism and is typically associated with elevated D-dimer levels, multifocal infarctions and advanced adenocarcinoma [1,2 ].

In up to 5–10% of patients with embolic stroke of undetermined source (ESUS), an occult malignancy is subsequently identified [3–5]. Although direct tumour embolism is considered rare, likely accounting for less than 1% of CAS, its true prevalence remains uncertain because histopathological examination of retrieved thrombi is not routinely performed in many centres after mechanical thrombectomy [6–8]. Lung cancer, particularly when invading the pulmonary veins, may provide direct access of tumour fragments to the systemic arterial circulation and has been reported as a rare cause of cerebral tumour embolism [9,10 ].

We report a case of acute ischaemic stroke in which histopathological examination of a thrombus retrieved during mechanical thrombectomy led to the diagnosis of previously unrecognised lung adenocarcinoma.

## Case presentation

A 76-year-old White European man with a history of tobacco use and no relevant family history of cancer or stroke presented with acute-onset left-sided hemiplegia, hemianopia and dysarthria. On admission, neurological examination revealed a National Institutes of Health Stroke Scale (NIHSS) score of 18.

**Fig. 1:**
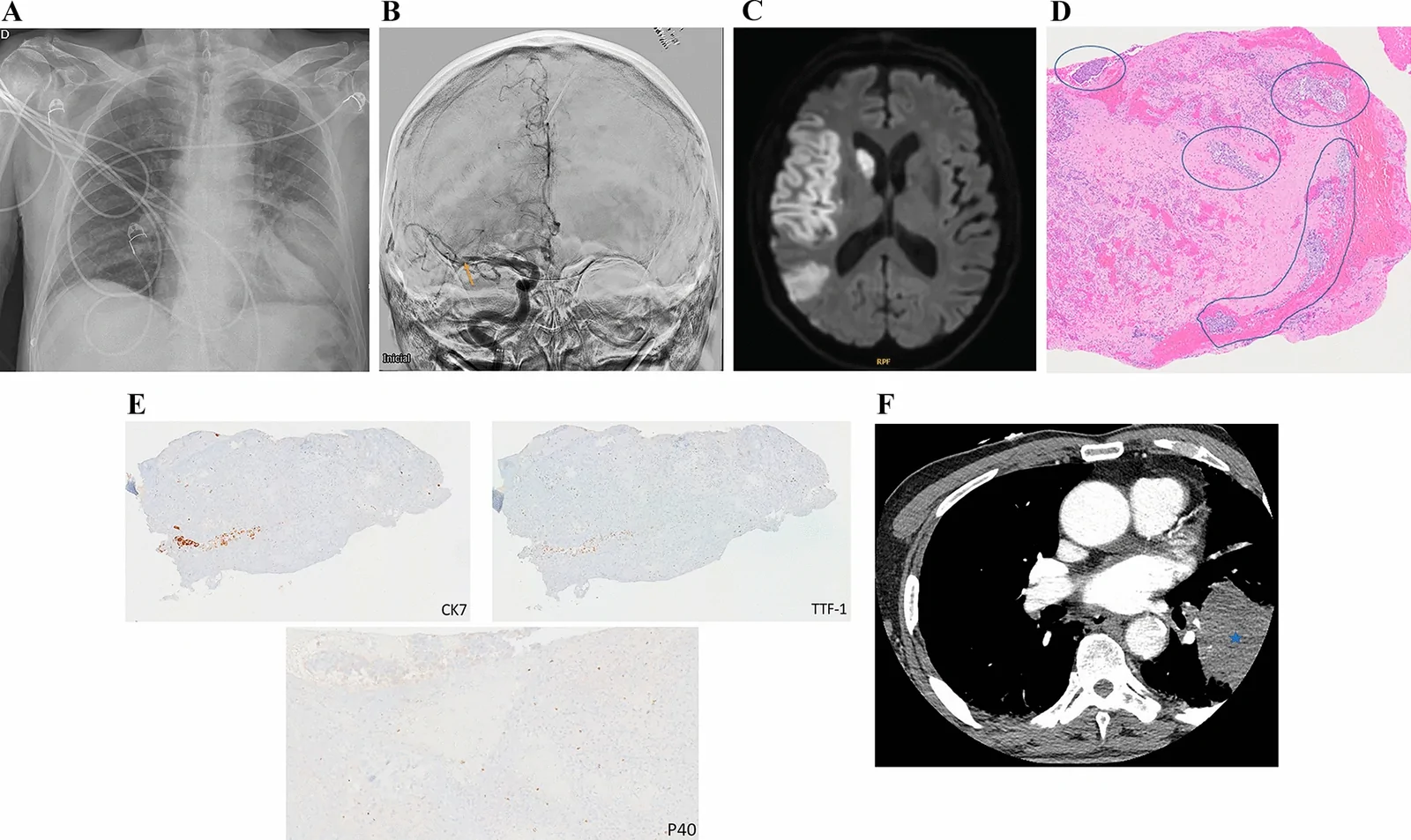
**A** Pre-treatment chest radiograph demonstrating a left retrocardiac opacity with non-specific features that did not allow diagnosis of the underlying pulmonary malignancy. **B** Digital subtraction angiography obtained at the beginning of mechanical thrombectomy demonstrating occlusion of the right middle cerebral artery. The orange arrow indicates the site of middle cerebral artery occlusion. **C** Diffusion-weighted brain MRI obtained 2 days after mechanical thrombectomy, demonstrating extensive acute infarction within the right middle cerebral artery territory without evidence of brain metastases. **D** Histopathological examination of the retrieved thrombus (haematoxylin and eosin staining) showing nests of malignant epithelial cells. The blue circles highlight the nests of malignant epithelial cells within the thrombus. **E** Immunohistochemistry consistent with poorly differentiated lung adenocarcinoma, including CK7 positivity, patchy weak TTF-1 expression, and negative staining for P40 and synaptophysin. **F** Thoracic CT obtained 5 days after mechanical thrombectomy, demonstrating a heterogeneous left lower lobe mass (9 × 7 × 8 cm) with central necrosis and invasion of the pulmonary vein. The blue asterisk indicates the primary lung mass

Non-contrast CT demonstrated early ischaemic changes with an Alberta Stroke Program Early CT Score (ASPECTS) of 7. CT angiography demonstrated occlusion of the right middle cerebral artery and CT perfusion showed a substantial penumbral area with a favourable mismatch profile. Intravenous thrombolysis was not administered because of partially established infarction. Before mechanical thrombectomy, no clinical or laboratory findings suggested an underlying malignancy or direct tumour embolism. Pre-treatment chest radiography demonstrated a left retrocardiac opacity; however, its radiographic appearance was non-specific and was not considered suggestive of an underlying pulmonary malignancy (Fig. 1A). Digital subtraction angiography performed at the beginning of the endovascular procedure confirmed the occlusion (Fig. 1B) and primary mechanical thrombectomy achieved partial recanalisation with a final modified Thrombolysis in Cerebral Infarction (mTICI) score of 2b.

Two days after mechanical thrombectomy, brain magnetic resonance imaging (MRI) confirmed extensive acute infarction in the right sylvian territory without evidence of brain metastases (Fig. 1C). Continuous cardiac telemetry and subsequent 72-h Holter monitoring showed no atrial fibrillation or other clinically relevant arrhythmias. Transthoracic echocardiography (TTE) showed no evidence of valvular vegetations, intracardiac thrombi or other major cardioembolic sources. Laboratory investigations were unremarkable except for moderately elevated D-dimer levels (1,476 ng/mL; reference range, 0–500 ng/mL).

Histopathological examination of the retrieved thrombus revealed nests of malignant epithelial cells, consistent with non-small cell carcinoma (Fig. 1D). Immunohistochemistry showed high programmed death-ligand 1 (PD-L1) expression (tumour proportion score, 90%), while next-generation sequencing identified a pathogenic *TP53* exon 5 mutation without potentially actionable alterations in *EGFR, BRAF, ALK, ROS1, KRAS, RET, MET, ERBB2, NTRK1, NTRK2* or *NTRK3*. Immunohistochemistry also demonstrated cytokeratin (CK7) positivity, patchy weak thyroid transcription factor-1 (TTF-1) expression, and negative staining for P40 and synaptophysin, supporting the diagnosis of poorly differentiated pulmonary adenocarcinoma (Fig. 1E).

Subsequent thoracic CT identified a heterogeneous left lower lobe mass measuring 9 × 7 × 8 cm, with central necrosis, mediastinal lymphadenopathy and invasion of the pulmonary vein (Fig. 1F).

The tumour was staged as cT4N2M1c (stage IV) poorly differentiated pulmonary adenocarcinoma. In the absence of actionable driver alterations and given the high PD-L1 tumour proportion score (90%), first-line treatment with pembrolizumab was initiated. The patient experienced partial neurological improvement and was discharged with mild residual deficits (NIHSS 7, modified Rankin Scale score 2).

Thirty days later, he was readmitted with fever, behavioural changes, left-sided sensory impairment and hyperphagia. Brain imaging demonstrated new frontal metastases. Despite ongoing treatment, his neurological and systemic condition deteriorated rapidly, and he died 51 days after the index stroke.

## Discussion and conclusions

This case illustrates a rare mechanism of CAS caused by direct tumour embolism from lung adenocarcinoma invading the pulmonary vein. Although hypercoagulability represents the predominant mechanism of CAS, direct tumour embolism is considered exceptionally rare, accounting for less than 1% of reported cases [6–8].

CAS represents a diagnostic challenge, particularly when ischaemic stroke precedes the diagnosis of malignancy [11]. Up to 5–10% of patients with ESUS are subsequently diagnosed with an occult cancer [4,5,12 ], most commonly adenocarcinomas, especially of pulmonary or pancreatic origin [3,13 ].

Several findings strongly supported direct tumour embolism in our patient. Histopathological examination demonstrated malignant epithelial cells within the retrieved thrombus, thoracic CT imaging identified direct pulmonary vein invasion by the primary tumour, and no alternative embolic source was identified despite prolonged cardiac monitoring and TTE. Pulmonary vein invasion provides a direct anatomical route for systemic tumour embolisation without requiring intracardiac shunting [9].

Representative published cases are summarised in Table 1. Consistent with previously reported cases, our patient had advanced pulmonary adenocarcinoma with pulmonary vein invasion. However, unlike most published reports, histopathological examination of the retrieved thrombus provided the first diagnostic clue leading to the diagnosis of an otherwise occult malignancy. This finding highlights the complementary diagnostic value of thrombus histopathology by prompting immediate investigation for an underlying malignancy, which in our patient led to thoracic imaging, oncological assessment and tumour staging, ultimately enabling timely initiation of systemic treatment.

**Table 1:** Representative published cases of cerebral tumour embolism secondary to primary lung malignancies and comparison with the present case

Author (year)	Age/Sex	Histology	Proposed embolic mechanism	Occluded artery	Neurological presentation	Acute reperfusion therapy	Histopathological findings in thrombus	Clinical outcome
Imaizumi et al. (1995) [10]	64/M	Adenocarcinoma	PV invasion	Right MCA	Acute ischaemic stroke	None	Tumour cells identified within the thrombus	Death
Inra et al. (2015) [14]	67/F	Adenocarcinoma	PV invasion	Not reported	Acute ischaemic stroke	None	Tumour cells identified within the thrombus	Death
Takasugi et al. (2017) [15]	62/M	Adenocarcinoma	Not demonstrated. Pulmonary metastatic tumour emboli	Multiple cerebral arteries	Recurrent ischaemic stroke	None	Tumour cells identified within the thrombus	Recurrent stroke; death
Pop et al. (2018) [16]	56/M	Sarcomatoid carcinoma	PV invasion	ICA/BA	Recurrent acute ischaemic stroke	MT	Tumour cells identified within the thrombus	Neurological improvement; death from progressive malignancy
Lemus et al. (2019) [17]	64/F	Poorly differentiated adenocarcinoma	PV and LA invasion	Left ICA terminus	Acute ischaemic stroke	MT	Malignant epithelial cells identified within the thrombus	Neurological improvement; death from progressive malignancy
Oyama et al. (2020) [18]	66/M	Pleomorphic carcinoma	PV invasion	Left ICA terminus	Acute ischaemic stroke	MT	Tumour cells identified within the thrombus	Neurological improvement; death from progressive malignancy
Moriyama et al. (2021) [19]	64/M	Squamous cell carcinoma	PV invasion	Right M2 MCA	Acute ischaemic stroke	MT	Tumour cells identified within the thrombus	Neurological improvement
Fujiwara et al. (2022) [20]	74/M	Squamous cell carcinoma	PV invasion	Left M2 MCA	Acute ischaemic stroke	MT	Tumour cells identified within the thrombus	Neurological improvement; death from progressive malignancy
Present case	76/M	Poorly differentiated pulmonary adenocarcinoma	PV invasion	Right M1 MCA	Acute ischaemic stroke	MT	Malignant epithelial cells identified within the thrombus (CK7 +, weak patchy TTF-1 +, P40 −, synaptophysin −)	Neurological improvement; death from progressive malignancy

M: male, F: Female, PV: pulmonary vein, LA: left atrial, MCA: middle cerebral artery, ICA: internal carotid artery, BA: basilar artery, MT: mechanical thrombectomy

Representative published cases were selected to illustrate the main clinicopathological features of cerebral tumour embolism secondary to primary lung malignancies and are not intended to represent a systematic review of the literature.

In our institution, all thrombi retrieved during mechanical thrombectomy are routinely submitted for histopathological examination as part of the standard pathological evaluation of extracted tissue. Although routine pathological assessment of thrombectomy specimens cannot currently be recommended for routine use, it may provide incremental diagnostic information in selected patients with ESUS, atypical clinical features, or when conventional investigations fail to identify an embolic source [7,8 ].

The patient showed only moderately elevated D-dimer levels. Although markedly elevated D-dimer concentrations are characteristic of hypercoagulability-mediated CAS, tumour embolism may occur despite normal or only mildly elevated D-dimer levels because the embolic material consists predominantly of malignant cells rather than fibrin-rich thrombus [15]. Therefore, normal or moderately elevated D-dimer levels should not exclude tumour embolism.

NBTE remains an important differential diagnosis in patients with CAS [21–23]. In the present case, no evidence of valvular vegetations was identified on TTE and prolonged cardiac rhythm monitoring demonstrated no atrial fibrillation. However, transoesophageal echocardiography (TEE) was not performed; therefore, occult NBTE cannot be completely excluded.

The optimal management of tumour embolism remains uncertain^24–26^. In contrast to hypercoagulability-mediated CAS, in which anticoagulation is frequently recommended, the role of anticoagulation in tumour embolism is unclear because the embolic material consists predominantly of malignant cells rather than fibrin-rich thrombus. Currently, there is insufficient evidence to guide specific antithrombotic strategies in this setting.

This report has several limitations. First, the absence of TEE precludes definitive exclusion of occult NBTE; therefore, a concomitant cardioembolic mechanism cannot be completely excluded. Second, this is a single-case observation and therefore does not allow conclusions regarding prevalence or optimal management. Finally, detailed quantitative analysis of thrombus composition was not available.

In conclusion, histopathological examination of the retrieved cerebral thrombus provided clinically relevant diagnostic information that contributed to the diagnosis of occult lung adenocarcinoma. Direct tumour embolism should be considered in selected patients with ESUS or suspected CAS, even in the absence of markedly elevated D-dimer levels. Pathological assessment of thrombectomy specimens may improve recognition of rare stroke mechanisms and facilitate earlier diagnosis of occult malignancy.

## Patient perspective

The patient´s family provided consent for publication and considered that sharing this case could contribute to improved recognition of uncommon mechanisms of cancer-related stroke.

## Learning points

- Cancer-associated stroke may be the first manifestation of occult malignancy, even when conventional diagnostic investigations are initially unrevealing.
- Direct tumour embolism should be considered in ESUS despite only moderately elevated D-dimer levels.
- Histopathological examination of thrombectomy specimens may provide clinically relevant diagnostic information by identifying uncommon stroke mechanisms, including occult malignancy.
- Pulmonary vein invasion by lung adenocarcinoma is an important mechanism of systemic tumour embolisation.

## Data Availability

No datasets were generated or analysed during the current study.

## Abbreviations

CAS: Cancer-associated stroke
ESUS: Embolic stroke of undetermined source
CT: Computed tomography
ECG: Electrocardiography
MRI: Magnetic resonance imaging
NIHSS: National Institutes of Health Stroke Scale
ASPECTS: Alberta Stroke Program Early CT Score
mTICI: Modified Thrombolysis in Cerebral Infarction
mRS: Modified Rankin Scale
TTE: Transthoracic echocardiography
TEE: Transoesophageal echocardiography
CK7: Cytokeratin 7
TTF-1: Thyroid transcription factor-1
PD-L1: Programmed death-ligand 1
NBTE: Non-bacterial thrombotic endocarditis
